# Reply to Prof. Joynes’ Paper on “Quarantine by the General Government,” and to Prof. Chaille on “Castration and Spaying as a Means of Arresting Syphilis”

**Published:** 1880-03-20

**Authors:** Alban S. Payne

**Affiliations:** Professor of Practice, in Southern Medical College


### Southern Medical Record.

EDITORS:

T. S. POWELL, M.D. W. T. GOLDSMITH, M.D. R. C. WORD, M.D.

*R. C. WORD, M.D., Managing Editor.*

☞All Communications and Letters on Business connected with the RECORD must be addressed to the Managing Editor.

VOL. X. ATLANTA, GA., MARCH 20, 1880. No. 3.

### ORIGINAL AND SELECTED ARTICLES.

### *REPLY TO PROF. LEVIN S. JOYNES' PAPER ON "QUARANTINE BY THE GENERAL GOVERNMENT," AND TO PROF. CHAILLE ON CASTRATION AND SPAYING AS A MEANS OF ARRESTING SYPHILIS.*

BY DR. ALBAN S. PAYNE,
Professor of Practice, in *Southern Medical College.*

[Continued from February Number.]

*Cold Water.*—Its free use externally with borax (the very best soap), would be found a sure preventive of a large number of those diseases that the physician is called upon to treat in low, wretched, over-crowded huts often found in large cities. At the same time the value, gratification and comfort experienced by a cool drink of water cannot be possibly over-estimated.

*Aconite.*—Of great value as a preventive of pain. It is a lowerer of the heart's action of very great power and certainty; is exceedingly valuable in the early stage of pneumonia, pleuro-pneumonia, inflammatory rheumatism, and in all diseases when it is desired to lessen the vis a tergo.

*Veratrum Viride.*—Very similar in its action to aconite on the system, with the exception that veratrum viride may run off by the bowels; aconite never does. For this reason aconite is the most reliable remedy in diseases which may have a tendency to assume an asthenic or typhoid type; worthy of a trial as an abortive remedy in the pleuro-pneumonia of cattle, in the stage of incipiency.

*Chlorate potassa.*—As a preventive remedy in hog-cholera (so called), with clean beds, warm, dry shelter, and some attention given to the condition of the hog's skin, along with a good nourishing diet, this remedy will be found reliable as a preventive and a curative agent in the hog cholera.

*Pulvis Cubebœi et Carbonate Iron.*—These two simple remedies will be found peculiarly beneficial in preventing and curing chicken-cholera.

*Vaccination* with the vaccine matter from the cow, as an aborting remedy in typhoid fever and the eruptive fevers generally, as suggested by Dr. R. L. Barret, Louisa county, Va.

As to the lavish use of quinine both as a prophylactic and curative means in yellow fever, during the past epidemic, it has either failed to meet expectation, or proved notoriously injurious. Even those outside of the profession are aware of this fact, and thereby are apt to place the faculty at a low estimate.

During the panic many resorted to the drug to the injury of their nervous system. One fact long ascertained, but apparently ignored, at present by many, is the following. The restoration of the secretions in this fever, and of course, by inference, the expulsion of the fever poison or its combinations by the channels of elimination, are true indications of treatment. Mercurials were used for this purpose (but not exclusively), and the "princes of thought" stopped short of salivation, so soon as the secretions were restored, whenever their aim could be attained without this risk. Patients rarely died when proper biliary evacuations were obtained, in this as in Asiatic cholera; this constituted a prognosis which should be treated as of great value. In this connection we therefore see that no board of health, without enlarged practical knowledge, assisted by an extensive acquaintance with the bibliography of yellow fever—such as belongs to men of enlarged reasoning powers, acute observation and ample training, can grapple with the subject successfully, or sift the wheat from the chaff.

Human life is too sacred to be subject to the mere vagaries of the imagination. On preventives, Bacon, *De Ang. Scient.*, says: *Neque dubitato inter desiderato reponere opus alaquid de curationibus morborum, qui habentes insanitibus.*

Although our profession reasoned by induction long before Bacon, yet this may be quoted for the benefit of Dr. Hand-Organ.

The instinct and conduct of animals in such matters, which closely resemble our constitution, may prove instructive to us. We must take into account the ability of the organism to resist, to a certain extent, physical influences.

Dyspepsia is often the forerunner of mental derangement. Do not overtask the stomach or brain.

Ignorance, improvidence and profligacy are the great evils of life.

Quarantine has so far never proved to be a success, and if it had proved itself to be what is claimed for it by its peculiar friends of the present day, still it is necessarily a subject of grave import, and like martial law, should not be declared without positive proof of its absolute necessity.

Unlike my learned friend, Prof. Joynes, I do not believe in the omnipotence of quarantine.

In my opinion, all fevers are a unit, either modified or intensified by atmospheric conditions. Let the thermometer run up to 98° in the shade, and remain there for some time, and let there be a want of cleanliness in a city, and you may calculate with a reasonable certainty that yellow fever will pay that city a visit before many days pass by, and without any importation from a foreign source. The same atmospheric conditions, only in a less intensified degree, and you may expect the prevalence of a remittent bilious fever. Nor am I one of those who believe you can confine in walls the pathogenic poison of yellow fever, as you could pen a hog or stall a cow. Philadelphia was a long time quarelling with and establishing quarantine against New York, on account of the yellow fever. At last wise counsels prevailed; she went to work and cleaned her own streets, and she has never since been visited by yellow fever. I well remember the calculation made at the South during the late war, that no federal army could summer in New Orleans; if it attempted to do such a thing their ranks would be decimated by the yellow fever. Gen. Buttler took command, he was not learned in medical science, but he possessed good, hard common sense and a practical turn of thought. He cleaned up the city thoroughly, and no case of yellow fever occurred. These two examples should be instructive and sure to teach us that cleanliness is one of the most valuable of preventive medicines.

I have read Prof. Joynes' argumentative paper entitled the "Need of a uniform National Quarantine" with a great deal of gratification as with much instruction. But with all due deference to his superior age and experience, I must say I think he mistakes the question at issue. It is neither the power of congress, nor the property of congress, to enforce a uniform, national quarantine law that is doubted by the masses; or rather can congress or any other set of men pass a law that can prevent the epidemic diseases from spreading? I am sure they cannot, unless they can temper the wind to blow wherever they listeth, or can regulate the temperature of the atmosphere at a point much below 98° in the shade.

My esteemed friend, Prof. Levin S. Joynes, in his paper "On the Need of the National Quarantine," says: "As to the question of expediency I am not deterred by any apprehension of danger likely to result from this enlargement of the operations of the general government, seeing that the new exercise of power will be for the safety and advantage of the entire country, more especially of the South, and it is not within the range of reasonable probability that it can ever be perverted to the purposes of injury or oppression."

This is a very graceful surrender of States Rights, the doctrine a long time held and very warmly cherished by the Professor and his political friends. Nevertheless a surrender not very emphatic in its terms, and made to dire necessity and with evidently some little qualms of conscience.

Now, so far as I know, no one is opposed to the general organization of boards of health in the several states; nor does any one object to the establishment of a national board of health or quarantine, but every physician having the love of science in his bosom and the true interest of the profession in his heart, does seriously object to their being used as huge engines of politico-medical power. For my own part I do not feel now, nor did I ever feel any very great solicitude about the fate of States Rights. I now believe and have always believed that a state stands to the general government as a county does to the state. Now what would some of Prof. Joynes' medico-political friends say were the County of Fauquier to refuse to obey, to nullify a law of the State of Virginia? And not being permitted to do this, were to secede? Why, the answer I conceive would come back quickly and in thundering tones : "Freeze her out! Stamp her out!"

I can quote the great names of a Patrick Henry, a John Marshall and a William West to sustain my views on these questions.

Nor am I one of those to be frightened by the stale dogmatical assertion periodically so common in sections of our common country, in regard to the dangers of imperialism. State medicine and States Rights ought to be, from the similarity of their name, twin brothers working in perfect harmony one with the other. My doctrine is to cure or to alleviate pain if constitutional, and, if unconstitutional, to use every effort, every means in my power to produce the same results. For I believe that (by divine right) this curing, this alleviating of suffering to the poor fallen human race is higher, nobler than aught in this world of ours, and, therefore, does and should override all constitutions and all and every consideration of public policy whatever. Besides, my religion, learned in my tender years at the lap of a sainted mother, teaches me "to do unto others as you would have others do unto you." I had read so much for the last few months about state medicine, preventive medicine, so much of which annoyed rather than interested me, for I could neither applaud or agree with much that I saw was being recommended to the general government to prevent visitations of epidemic diseases, such as "freezing out" the contagion or placing it in close confinement to the yellow fever contagia as the sole means of preventing its spread. The "stamping out" process also to be adopted in regard to all cases afflicted with pleuro-pneumonia in its benign form or stage to prevent rinderpest. The same process to be adopted with hogs to prevent an epidemic of hog cholera; decapitating chickens to prevent chicken cholera. Even such to be followed by the recommendation to "stamp out" all horses found affected with "farcy" to prevent the "glanders," until I was in such a frame of mind that I hardly thought any recommendation, however absurd or monstrous, would surprise me. But in this I was mistaken, for I was greatly surprised to hear Prof. Chaille, of New Orleans, read a paper to the American Medical Association at Atlanta, Ga., May the 6th, 1879, in which he urged the general government to strictly enforce by penal enactment, the *castration* of the men and the *spaying* of the women to prevent the spread of syphilis. His paper was very artistically read, and was beautifully worded, evincing considerable dramatic power and literary taste and ability. It was a pity and a shame for so elegant a paper to have been ruined by so monstrous a proposition. Carry out this idea of Prof. Chaille and our government would cease to be the

"home of the brave and the land of the free" and become the most cruel oligarchy on the face of earth.

Now I would pass this paper over as one of the vagaries of a bright, active, over-worked brain, were it not from the fact that this particular recommendation was received with great applause by some members of the National Board of Health.

And if I am correct in this belief, sincerely (although it may be erroneously entertained), it becomes a significant fact, and one well calculated to cause grave solicitude for the peace and quiet of our country.

Now this theoretical recommendation sounds well enough on paper, but the practical working of it must be just the reverse of what the learned Professor supposes. And, in the first place, the castrated male would be the subject of almost continual exacerbations, sexual desire, and he would be perpetually on the *qui vive* for some female subject to relieve his sexual desires upon. Consequently he would be engaged in sexual intercourse with a much larger congress of women than would naturally be desired by a man of virility. Therefore the risk of his spreading the disease would be far greater in him than the risk of an unemasculated man would be.

There is, mind you, nothing in simple castration preventive of syphilis. In circumcision there would be some increased chance of exemption from contracting syphilis, very decidedly so in gonorrhœa. The loss of the testicles does not abate the desire for sexual commerce in man, but only prevents its proper and satisfactory consummation. The ancient eunuch was a man who had lost the penis as well as both testicles. The man who had lost one testicle in ancient times was called *spodanes*.

The female also burning with a sense of injustice, coupled with a sense of degradation, would be continually brooding over her great wrong, and naturally anxious to prove the operation of the learned Doctor a failure, so far as she herself was concerned. She would be seeking every opportunity to convince as many males as possible of this fact, until, lost to shame, she would become a common prostitute. This thing would never be willingly submitted to and would have to be enforced by the "strong arm of the law," and thereby you would throw upon every community a large class of malcontents who would render every peaceful community a perfect pandemonium.

I may state that the worse man I ever saw to *run* after women was a large handsome fellow who had been castrated, ten years before, in Danville, Kentucky, by my uncle, the late Dr. Alban Goldsmith. He had sexual intercourse with more women, talked more of his virile powers, and gave more of his paramours the syphilis than any man I ever met with. After spending his own property, late in life, he married a lady with some property of her own. There was no offspring following this marriage, and so far as I know, he succeeded in fooling his wife to the last. However, his wife was heard once only to remark, about a year after this marriage, that Mr. G. could go to bed earlier and to sleep quicker, and get up earlier in the morning than any man she ever heard of. Of course, whenever one of those emasculated men marry, their whole inclination is to go from home.

And here I am reminded of what actually occurred with an old lady in Southwest Virginia. Her husband had the mumps, and it became necessary to send for Dr. F——. The doctor, not knowing what the disease was, was met at the gate by the old woman, who was very much excited and quite solicitous regarding "her old man," exclaiming : "Doctor, he has got the mumps and they have *fell*, and I reckon they are nigh unto this large," extending her double fists directly in the doctor's face to give him some idea of the size. "Now, Doctor, what can be done ?" exclaimed the old woman. The doctor replied with an oath, that if he could not cure him he knew what he could do.

"What?" exclaimed the old woman.

"Why, cut them out."

"Goodness!" exclaimed the old woman, "do everything *but that*. Yes, Doctor, let it be the very *lastest* thing, for you know they are the most *particularest thing* a man has."

